# Short- and Long-Term Epoxy Modification of Bitumen: Modification Kinetics, Rheological Properties, and Microstructure

**DOI:** 10.3390/polym12030508

**Published:** 2020-02-26

**Authors:** Antonio A. Cuadri, Clara Delgado-Sánchez, Francisco Javier Navarro, Pedro Partal

**Affiliations:** Departamento de Ingeniería Química, Centro de Investigación en Tecnología de Productos y Procesos Químicos (Pro2TecS), Campus de ‘El Carmen’, Universidad de Huelva, 21071 Huelva, Spain; antonio.cuadri@diq.uhu.es (A.A.C.); clara.delgado@diq.uhu.es (C.D.-S.); partal@uhu.es (P.P.)

**Keywords:** epoxy bitumen modification, bitumen processing, rheology, process rheokinetics, short-term modification, long-term modification

## Abstract

Aiming to gain knowledge in the epoxy-bitumen modification mechanisms, this work explores the effects that epoxy concentration and ambient curing exert on the physico-chemistry and thermo-rheological properties of epoxy-modified binders. Process rheokinetics of epoxy-bitumen blends indicates that binder short-term modification (i.e., during processing) is accelerated by epoxy concentration. Furthermore, a synergistic effect of epoxy concentration and ambient curing is found during long-term modification (i.e., during curing at ambient conditions). As a result, viscous and viscoelastic rheological properties of binders are enhanced at medium/high in-service temperatures, at least, after one month of curing. FTIR (Fourier Transform Infrared spectroscopy) tests and SARAs (Saturates, Aromatics, Resins and Asphaltenes) analysis confirm the existence of esterification/etherification reactions between epoxy oxirane groups and the carbonyl groups available in aromatic and resin molecules. Thus, the new high molecular weight compounds increase the asphaltenic fraction of modified bitumen. Likewise, nonreversing heat flow curves obtained by modulated calorimetry corroborate the formation of such highly structured domains responsible for the final binder performance.

## 1. Introduction

The drastic increase in vehicle traffic, load, and speed, along with weathering, may result in a shortening of the in-service life of asphaltic pavements [1]. Related to this, usual distresses, such as rutting (permanent deformations in pavements due to high temperatures) and thermal cracking (low temperature fractures due to asphalt poor flexibility), have been traditionally overcome by bitumen modification with virgin polymers (SBS, SBR, EVA, etc.) or polymeric wastes (plastic from agriculture, tire crumb rubber, etc.) [2,3,4,5,6,7]. This bitumen modification is achieved through a suitable dispersion of the polymer into the molten bitumen. In particular, high molecular weight modifiers require both high processing temperatures and high shear to avoid polymer/bitumen phase separation during binder storage at high temperatures without stirring [8,9,10]. Obviously, all of this causes a significant increase in both production costs and operators’ safety and serious operational limitations. 

With all this in mind, current pavement engineering research focuses on the exploration of new modification approaches in order to develop bituminous materials with enhanced processability, workability, and long-lasting characteristics [11]. In this sense, nonpolymeric reactive agents (e.g., acids, organic additives, isocyanate-functionalized polymers, or dioxide/thiourea-derivatives) have been also used in the paving industry, which are able to form chemical bonds with bitumen compounds [12,13,14,15,16,17]. 

On these grounds, although some experiences using epoxy modifiers were reported in the 1950/1960s [1,18], the modification technology with epoxies has recently attracted the attention of pavement scientists and technicians. Some papers [11,19,20,21] have reported that the addition of epoxy compounds to bitumen produces chemical crosslinking during their mixing stage, the extent of which strongly depends on the processing conditions (i.e., time and temperature) [19,20,21] and leads to thermoset bituminous binders with enhanced performance to rutting phenomena [11]. It can thus be stated that an adequate selection of the processing conditions (i.e., time and temperature) should enable epoxy-asphalt mix to be handled and compacted in a partially cured state. Afterwards, further crosslinking would continue at ambient temperature until the binder develops to full strength over two to four weeks [1]. In order to accelerate the crosslinking process of the bituminous materials, hardening agents have also been included in the formulation of the epoxy bitumen binder [11,19,22]. However, this brings with it an increase in costs and a greater risk of gelation.

Additionally, other works [19,22,23,24] have reported the use of epoxies as a rejuvenator of SBS bituminous binders. It is well known that the degradation of SBS affects the binder’s mechanical properties. In this sense, a valuable restoration of the SBS network structure contained in SBS binders can be achieved thanks to the reactions between epoxy functional groups with the carboxylic groups formed in the degradation of SBS. 

Therefore, although some attempts have been made to use epoxies for bitumen modification, a general lack of knowledge still exists. Thus, the novelty of this paper is to study the effects that epoxy addition to neat bitumen (together with ambient curing) exerts on the modification mechanisms, microstructure, and physicochemical characteristics of the resulting bituminous binders. 

With this aim, different amounts (from 0.5 to 4 wt %) of an epoxy modifier (bisphenol A diglycidyl ether) were added to neat bitumen. These low concentrations of epoxies were selected to avoid the gelation phenomena. Bitumen-epoxy modification kinetics during the mixing process (short-term modification) and curing stage (long-term modification) were studied by rheokinetics tests, rheological measurements, modulated calorimetry (MDSC), thin-layer chromatography (TLC-FID), and infrared spectroscopy (FTIR).

## 2. Experimental

### 2.1. Materials

A bitumen with 61 dmm penetration and 50.2 °C ring-and-ball softening temperature (EN 1426:2015 and EN 1427:2015) was used as base material for the bitumen modification. The selected epoxy modifier was Bisphenol A diglycidyl ether (hereinafter referred to as BPA), a bifunctional epoxy with a molecular weight of 340 g·mol^−1^ and epoxy value of 0.52 mol/100 g, supplied by Panreac, S.A. (Panreac Química, Barcelona, Spain).

### 2.2. Modified Bitumen Processing

The modification kinetics during the mixing process was studied by recording binder torque evolution (related to its viscosity) with time, in a controlled-stress rheometer RS-600 (Haake, Thermo Electron GmbH, Karlsruhe, Germany). An open batch mixer was used, which consisted of a cylindrical vessel (42 mm diameter, 88 mm height) and a helical ribbon impeller (38 mm diameter). To that end, 90 g of base bitumen was added to the vessel and introduced in an oven for 20 min at the processing temperature (180 °C). Afterwards, the vessel was placed in the rheometer and the helical ribbon impeller was moved to 3 mm above the bottom of the vessel, allowing an agitation speed of 90 rpm. After 10 min mixing, epoxy was added at concentrations between 0.5 and 4 wt %, and the blend was stirred for 24 h. After the 24 h processing, the resulting binder was divided into two parts: one was tested as such (these samples will be referred to as noncured binders), and the other was exposed to ambient conditions for one month (the so-called cured binders). 

For the sake of comparison, base bitumen was also processed under the same mixing conditions (90 rpm, 180 °C, and 24 h), obtaining the so-called blank sample. In addition, a portion of the blank sample was also subjected to the same ambient curing. Finally, a reference bituminous sample containing 3 wt % SBS triblock copolymer (Kraton D-1101 with 31 wt % styrene content and, respectively, weight and number average molecular weights of 1.5 × 10^5^ and 8.98 × 10^4^ g·mol^−1^) was prepared with a Silverson L5M homogenizer at 180 °C for 2 h.

### 2.3. Samples Testing

The rheological characterization was conducted on bituminous binders using a controlled-stress rheometer Physica MCR-301 (Anton Paar, Ostfildern, Germany) and a plate-plate geometry (1 mm gap and 25 mm diameter). Different rheological measurements were carried out: (a) on the one hand, viscous flow tests at 60 and 135 °C; and (b) on the other hand, temperature sweep tests from 30 to 100 °C (1 °C·min^−1^ heating ramp), performed in oscillatory shear at 10 rad/s and a strain within the linear viscoelastic range. In order to ensure accurate rheological results, every sample was measured at least twice. 

Thin layer chromatography coupled with a flame ionization detector (TLC/FID) was used for the determination of SARAs fractions (i.e., saturates (S), aromatics (A), resins (R), and asphaltenes (As)) of blank sample and epoxy-modified binders. Tests were conducted in an Iatroscan MK-6 analyzer (Iatron Corporation Inc., Tokyo, Japan). Elutions were performed with hexane, toluene, and dichloromethane/methanol (95/5) [25].

Fourier transform infrared spectroscopy (FTIR) spectra of binders were obtained with a Jasco FT/IR 4200 spectrometer (Jasco Analytical Instrument, Tokyo, Japan). Firstly, a weight of 0.7 g of blank sample or epoxy-modified binders was dissolved in 25 mL dichloromethane. Afterwards, the solution was always placed on a potassium bromide disk (32 mm × 3 mm) and exposed to ambient for solvent evaporation. Finally, the KBr disk was laid into the sample holder, and the FTIR spectra were obtained in a wavenumber window of 400–4000 cm^−1^ at 4 cm^−1^ resolution in the transmission mode.

Modulated differential scanning calorimetry (MDSC) tests were conducted on bituminous binders with a TA Q-100 (TA Instruments, New Castle, DE, USA). Samples of 5–10 mg were always subjected to the following procedure: (a) a first heating scan from room temperature to 210 °C was carried out with the goal to remove the thermal history of the sample; (b) a cooling ramp, to −60 °C; and (c) a second heating ramp, to the same final temperature used for the first heating scan. The selected conditions were: heating/cooling rate of 10 °C·min^−1^, amplitude of modulation of ±0.5 °C, a period of 60 s, and a flow of 50 mL/min N_2_ as purge gas. The characteristic temperatures and enthalpies were determined during the second heating sequence.

## 3. Results and Discussion

### 3.1. Short- and Long-Term Rheological Modification

**Figure 1:**
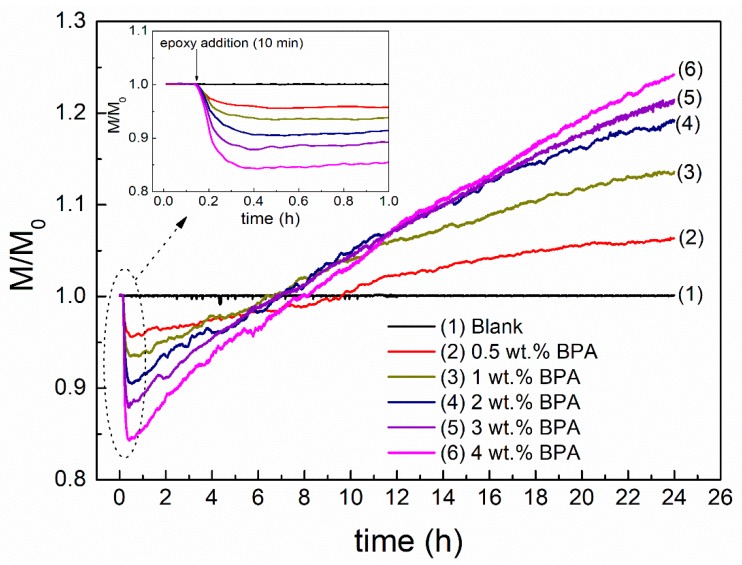
Evolution of relative mixing torque during processing as a function of the added epoxy concentration.

The kinetics of the short-term bitumen modification at 180 °C was assessed by monitoring the evolution of the normalized torque (M/M_0_) with mixing time. The value of M_0_ corresponds to the constant torque obtained before epoxy addition (which takes places after 10 min of mixing), and M is the actual torque for each mixing time. Thus, M/M_0_ allows us to quantify torque changes only due to the epoxy modification, regardless of the torque value just before epoxy addition (i.e., M_0_). Figure 1 displays the evolution of M/M_0_ for the base bitumen (which results in the blank sample after completing its processing) and their corresponding epoxy-modified binders containing different epoxy concentrations. 

First of all, it can be observed that base bitumen experienced negligible evolution of M/M_0_ after 24 h processing time, which points out that the selected processing conditions (180 °C for 24 h) does not produce a significant hardening in the binder due to oxidation. As for the epoxy-modified binders, all of them display the same pattern: (a) a first region with a constant value of M/M_0_ (equal to 1) before epoxy addition; (b) a marked decrease in M/M_0_ due to the plasticizing effect caused by the addition of a liquid with low viscosity, which becomes more pronounced as epoxy concentration increases; and (c) a monotonous increase in M/M_0_ with no trend to level off. This behavior is ascribed to an epoxy-induced bitumen hardening, or the chemical crosslinking, since the effect of bitumen oxidation was discarded with the result observed for the base bitumen. In addition, the value of M/M_0_ after completing processing, which corresponds to the final binder hardening, is proportional to the epoxy concentration. Thus, the highest value was observed for the binder prepared with 4 wt % BPA. 

Interestingly, the continuous increase of M/M_0_ curves shown in Figure 1, with no trend to a constant value, suggests all binders still have available epoxy groups for further crosslinking after 24 h mixing. Hence, an additional hardening is expected during their ambient curing process (long-term modification), as reported by Polacco et al. [1].

**Figure 2:**
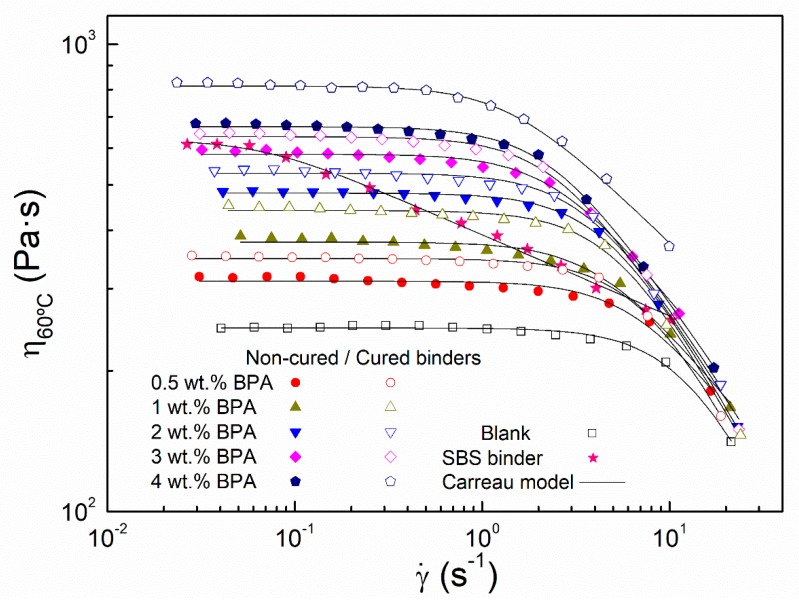
Viscous flow curves at 60 °C for noncured and cured epoxy modified binders. Comparison with a 3 wt % SBS-modified bitumen and neat bitumen subjected to the same mixing conditions (blank sample).

As a whole, short- and long-term epoxy modifications involve changes in binder rheological properties that affect its application in pavements. A rheological characterization based on viscous flow curves, at 60 and 135 °C, and temperature sweep tests were conducted on the binders just after 24 h processing (noncured binders) and on those exposed to ambient curing conditions for one month (cured binders). Figure 2 displays the viscous flow curves at 60 °C, which is considered as a characteristic in-service temperature for warm climates. 

As can be seen, all binders show the same viscous response characterized by a Newtonian zone at low shear rates, with constant values of viscosity, followed by a shear-thinning region above a critical shear rate. This viscous behavior can be fitted to the Carreau model:

(1) $$\frac{\eta }{\eta_{0}}=\frac{1}{{\left[1+{\left(\lambda \cdot \overset{\cdot }{\gamma }\right)}^{2}\right]}^{s}}$$

where $\eta_{0}$ is the zero-shear-rate limiting viscosity observed in the Newtonian zone, λ(s) is a characteristic time whose inverse value approximately matches the shear rate for the onset of the shear-thinning region, and ‘*s*’ is a parameter related to the slope of this region. Carreau model parameters for all binders are gathered in Table 1. 

**Figure 3:**
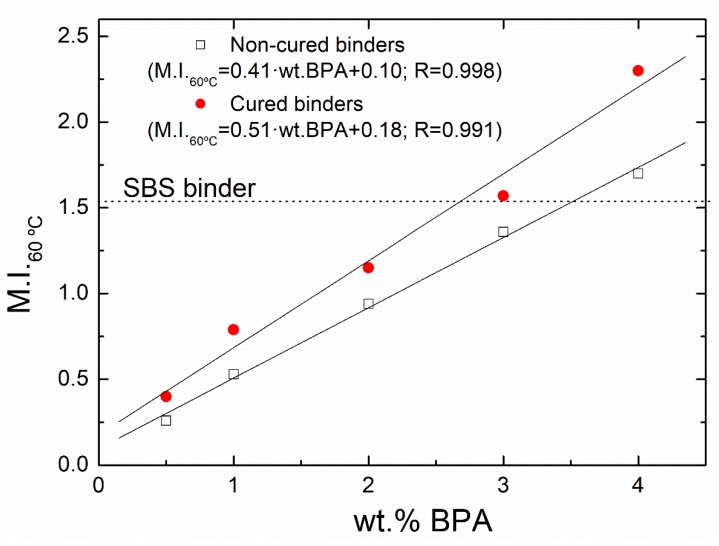
Evolution with epoxy concentration of the Modification Index (M.I._60 °C_) achieved at 60 °C for noncured and cured binders.

As deduced from Figure 2 and Table 1, a gradual increase in viscosity values are noticed in noncured binders as BPA concentrations rises, which is in line with the hardening (i.e., final values of M/M_0_ after 24 h processing) observed in Figure 1. Interestingly, the subsequent ambient curing of 24 h noncured binders produces an increase in their viscosity values. It is important to note that there is no increase in viscosity when the blank sample is subjected to the same ambient curing process. Therefore, these results clearly indicate that crosslinking of bitumen compounds, via epoxy reactions, continues after processing for at least one month. Moreover, results shown in Table 1 reveal that BPA addition to base bitumen (and the subsequent curing process) leads to a more complex microstructure (with larger values of λ), being bituminous materials a more sensitive to the application to shear stress [26], if compared to the blank sample. This fact is attributed to the formation of polymeric structures resulting from epoxy-bitumen crosslinking. In addition, zero-shear-rate limiting viscosities ($\eta_{0}$) gathered in Table 1 have been considered to quantify the bitumen modification degree at high in-service temperatures. To that end, a modification index (M.I._60 °C_) has been defined as follows:

(2) $$M.I._{60{}^{\circ}C}=\frac{\eta_{0,\mathrm{mod}}-\eta_{0,\mathrm{blnak}}}{\eta_{0,\mathrm{blnak}}}$$

where $\eta_{0}{}_{,\mathrm{mod}}$ is the zero-shear-rate limiting viscosity of epoxy-modified binders and $\eta_{0}{}_{,\mathrm{blank}}$ is that value corresponding to the blank sample, both of them at 60 °C. Figure 3 shows the evolution of this parameter with epoxy concentration for noncured (short-term modification) and cured (long-term modification) binders. 

As expected, a higher viscosity enhancement is noticed for the noncured binders as epoxy concentration rises, which is favored after their subsequent ambient curing process. Likewise, 3 wt % cured binder presents a higher modification degree than that found for the reference SBS-binder, which is also surpassed even for 4 wt % binder without curing process. Once again, the M.I._60°C_ differences between noncured and cured binders allow us to state that even after 24 h of processing there are epoxy groups available for further reaction with bitumen compounds during the ambient curing, this difference being more evident for the binder prepared with 4 wt % BPA. Moreover, $\eta_{0}$ is also considered an adequate parameter in predicting the resistance of paving binders to permanent deformation at high in-service temperatures [27,28]. Consequently, $\eta_{0}$ values (or modification indexes) indicate that, by controlling both processing and curing stages, it is possible to manufacture bituminous binders (and, therefore, asphalt mixes) with better performance to rutting than SBS-binder.

**Figure 4:**
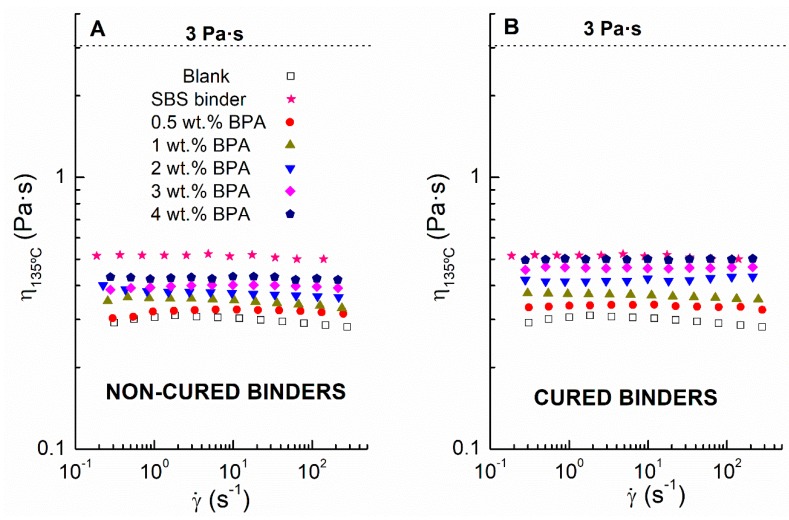
Viscous flow curves at 135 °C of (**A**) noncured and (**B**) cured epoxy modified binders. Comparison with a 3 wt % SBS-modified bitumen and neat bitumen subjected to the same mixing conditions (Blank sample).

Additionally, binder viscosity at 135 °C gives valuable information about its pumpability, mixability, and mix workability [29]. Figure 4 displays the viscous flow curves, at 135 °C, for noncured (Figure 4A) and cured (Figure 4B) binders, as well as for blank sample and SBS-reference binder. 

According to AASHTO MP320 standard, if binder viscosity at 135 °C exceeds 3 Pa·s, the asphalt mixtures become too hard to be compacted, and it is difficult to form a suitable pavement surface [19]. As seen, all binders present viscosity values between 0.3 and 0.5 Pa·s in the whole shear rate tested, therefore meeting the specification. 

**Figure 5:**
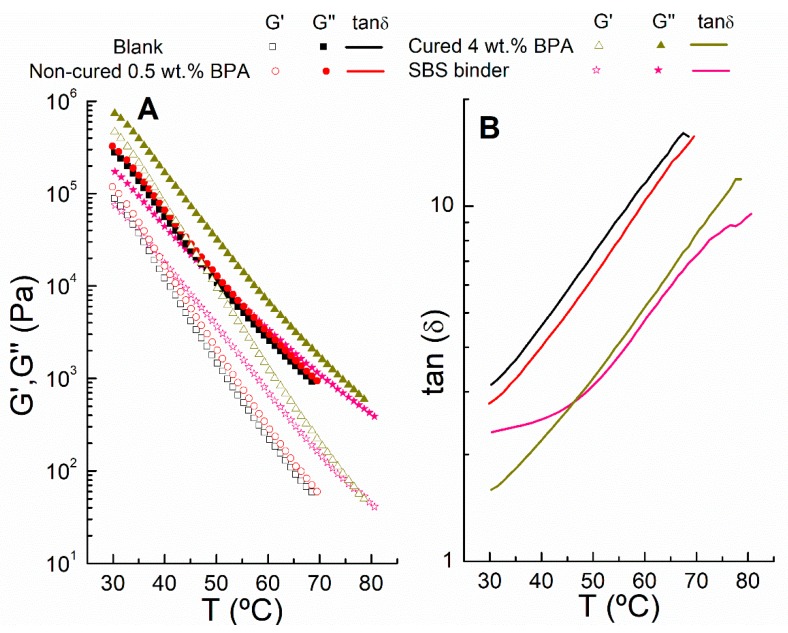
Dependence on temperature for selected binders of: (**A**) elastic (G’) and viscous (G”) moduli; and (**B**) loss tangent (tan δ = G”/G’).

Finally, oscillatory shear temperature sweep tests, from 30 to 80 °C, were conducted on the reference samples (blank sample and rubber SBS-binder) and two selected epoxy binders (noncured 0.5 wt % BPA and cured 4 wt % BPA). Thus, the evolution with the testing temperature of elastic (G’) and viscous (G’’) moduli and loss tangent (tan δ = G”/G’) are plotted in Figure 5A,B, respectively. 

All bituminous binders show a monotonous decrease of both moduli with increasing testing temperature, prevailing a viscous character (with values of tan δ > 1). As expected, the 0.5 wt % BPA binder, just after 24 h processing, presents a slight increase in G’ and G’’ with respect to the blank sample. However, 4 wt % BPA cured at ambient conditions displays a marked increase, with both moduli clearly higher than those observed for the SBS-binder (the difference being more evident at low testing temperature). This result is in line with the previous viscous flow response at 60 °C. 

In all cases, tan δ curves show the elastic binder characteristics decrease with testing temperature. Conversely, the epoxy bitumen modification with 0.5 wt %, without ambient curing, produces a decrease in tan δ values in the entire range of testing temperature (enhancing binder elastic properties). This effect is more pronounced for the cured binder prepared with 4 wt.% BPA. On the other hand, the slope of the tan δ vs. T curve is related with the binder thermal susceptibility at medium/high in-service temperatures. Thus, SBS-binder displays a reduced susceptibility to temperature in the interval of ca. 30–45 °C, with nearly constant tan δ values, similarly to that reported by Martin-Alfonso et al. [30]. As for epoxy binders and blank sample, even though they present a similar slope in their tan δ curves (and therefore no alteration in thermal susceptibility), cured 4 wt % BPA exhibits quite similar tan δ values in the medium/high in-service temperatures (45–70 °C) than the commercial SBS binder. 

### 3.2. Physicochemical Characteristics and Microstructure of Epoxy-Modified Bitumens

The above-commented changes in rheological performance of epoxy modified binders at high in-service temperatures are related to significant changes in their physicochemical characteristics and microstructure. In order to clarify this, FTIR, TLC/FID, and MDSC tests were performed on selected samples.

**Figure 6:**
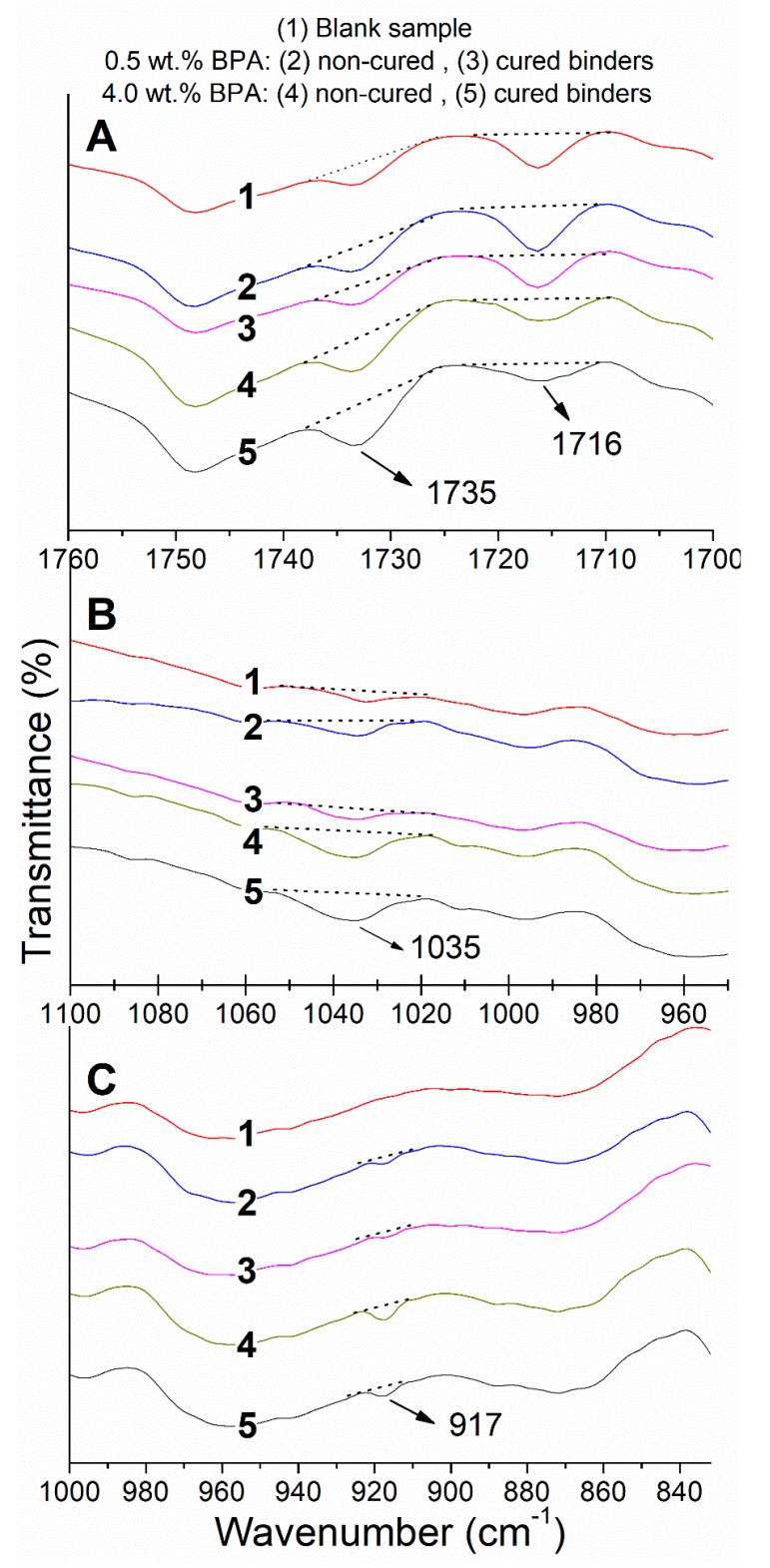
FTIR spectra for selected epoxy-modified binders and blank sample in three different intervals: (**A**) 1760–1700 cm^−1^, to evaluate carbonyl acid and ester groups at 1716 and 1735 cm^−1^, respectively; (**B**) 1100–950 cm^−1^, and (**C**) 1000–830 cm^−1^ to evaluate the ether and oxirane groups at 1035 and 917 cm^−1^, respectively. Dotted lines have been used to mark the corresponding peak area.

It has been reported that the oxirane (C_2_H_4_O) groups, which are generated after epoxy ring opening, react with the carbonyl acid groups (RCOOH) present in the bituminous phase forming ester (RCOOR’’) and ether (ROR´) compounds [11,19,24]. Thus, in order to assess the extent of this reaction due to both epoxy concentration and ambient curing, FTIR spectra of selected binders have been plotted in three different intervals in Figure 6: (A) 1760–1700 cm^−1^, to evaluate carbonyl acid and ester groups at 1716 and 1735 cm^−1^, respectively; (B) 1100–950 cm^−1^, and (C) 1000–830 cm^−1^ to evaluate the ether and oxirane groups at 1035 and 917 cm^−1^, respectively. 

As a whole, an increase in the reaction extent should be noticed as an increase of the areas corresponding to the bands at 1735 cm^−1^ (RCOOR”) and 1035 cm^−1^ (ROR’) together with a decrease of peaks at 917 cm^−1^ (C_2_H_4_O) and 1716 cm^−1^ (RCOOH). Accordingly, epoxy binder prepared with the highest epoxy concentration and subjected to ambient exhibits the largest marked areas for the reaction products (see peaks at 1735 and 1035 cm^−1^ in Figure 6A,B). This epoxy-binder also displays the smallest areas for the reactants (peaks at 917 and 1716 cm^−1^ in Figure 6A,C). As for the effects of epoxy concentration, if attention is paid to the noncured binders (curves 2 and 4), it is clear that a higher amount of epoxy promotes those reactions. Therefore, the enhanced rheological properties found in epoxy binders can be clearly related to the creation of the crosslinked network through new ester and ether groups. In addition, the peak still observed at 917 cm^−1^ for cured binders (curves 3 and 5 in Figure 6C) corroborates the presence of remaining oxirane groups (available for further reactions), even after a month of curing. Thereby, binder rheological properties are expected to improve for a longer period of in-service life.

On the other hand, due to the complexity of its chemical composition, bitumen has been broadly characterized by fractionation techniques (e.g., through thin layer chromatography, TLC/FID) into the so-called “SARAs” fractions (i.e., saturates, aromatics, resins, and asphaltenes), according to their solubility and polarity. Although still a matter of debate, different models have been proposed to describe how these fractions are arranged to constitute the bitumen’s microstructure [31]. Among them, the colloidal model is the most realistic to ascertain the effects of esterification and etherification reactions exerted on bitumen’s behavior at high in-service temperatures. In accordance with this model, micelle-like structures formed by asphaltenes agglomerates are surrounded by high-molecular weight polar compounds (mainly resins) dispersed in a maltene fraction composed by saturates, aromatics, and the rest of resins [1,32,33,34]. Thus, micelle sizes and the relative proportions of these fractions due to the arrangement of polar bitumen compounds strongly affect the thermo-rheological behaviour of bitumen, and it is accepted that more structured systems display higher viscosity [35,36]. 

**Figure 7:**
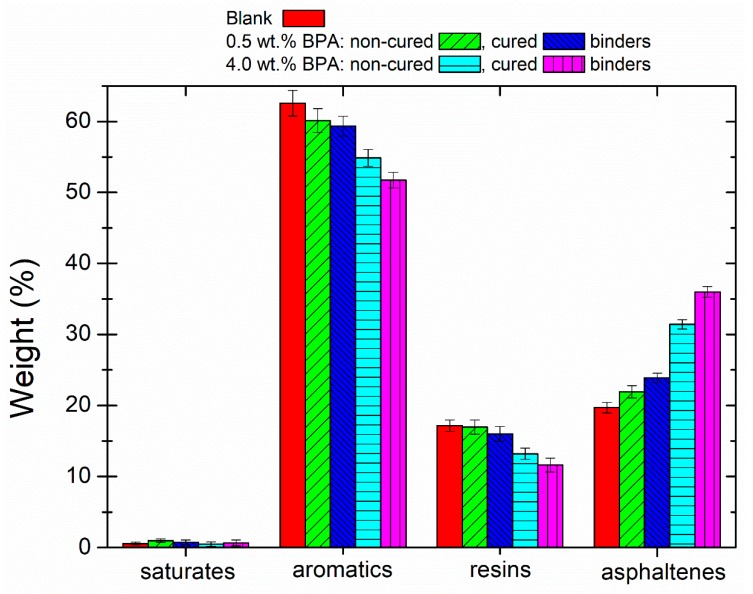
SARAs fraction obtained by TLC-FID of selected epoxy-modified binders and blank sample.

As seen in Figure 7, whilst all samples display a similar content in saturates, due to their low chemical reactivity, significant variations are noticed in the other fractions. When epoxy content in binders is raised (or they are subjected to ambient curing) the aromatics and resins fractions decrease, giving rise to a significant increase in the asphaltenes. Thus, compared to the blank sample, the cured 4 wt % BPA binder presents reductions of 10% aromatics (from 62 to 52 wt %) and 6 wt % resins (from 17 to 11 wt %), which are accompanied by a rise in the asphaltenes of 16 wt % (from 20 to 36 wt %). These results indicate that the esterification and etherification reactions take place between the oxirane groups and high molecular weight molecules found in aromatics and resins that contain carbonyl acid groups. The new bitumen compounds formed would not be eluted during the chromatographic separation, and therefore, they are quantified in the asphaltenes content [33,35,37]. It is evident that the development of more complex structures agrees with the above-commented rheological results. 

Delving deeper into the microstructure, MDSC conducted on these selected binders also provides valuable information. As reported by Masson et al. [38,39], four events can usually be observed in the nonreversing heat flow curves when the different bitumen fractions reorder with increasing temperature: a first event characterized by a broad endothermic background located from −30 to 85 °C; two exothermic events typically centered at ca. −5 and 25 °C; and the fourth event, an endotherm from 25 to 80 °C. 

**Figure 8:**
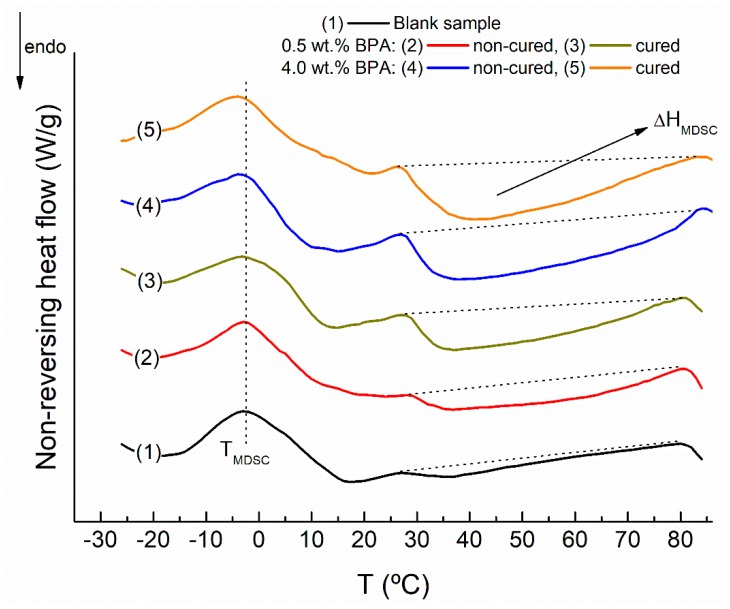
Nonreversing heat flow curves, from modulated calorimetry (MDSC) tests, for selected epoxy-modified binders and blank sample.

As depicted in Figure 8, noncured 0.5 wt % BPA binder displays a significant increase in the area corresponding to the fourth endothermic event (associated to an enthalpy, ΔH_MDSC_), being greater after its subsequent ambient curing. This effect is much more pronounced for those binders prepared with the highest epoxy concentration (Figure 8 and Table 2). The fourth event is ascribed to the melting of relatively large (or high molecular weight) compounds, as those normally found in asphaltenes that reorder forming mesophasic domains [38,39]. As seen in Figure 7, chemical reactions between oxirane and the carbonyl groups led to higher molecular weight compounds that increased the asphaltenic fraction. These more structured and ordered mesophasic microstructures need more heat for their melting process and are responsible for the observed increase in ΔH_MDSC_ values (Table 2).

On the other hand, the second event is provoked by a time-dependent cold crystallization of low molecular weight saturated segments [38,39] and appears at a characteristic temperature (T_MDSC_). Lower values of T_MDSC_ shifts chain mobility to lower temperatures (i.e., lowering the temperature at which crystallizable segments can crystallize) [38,39,40]. Compared to blank sample, although no significant differences are noticed for noncured 0.5 wt % BPA binders, T_MDSC_ gathered in Table 2 is shifted to a lower value for the binder containing 4 wt % BPA and, particularly, after ambient curing. This result would indicate an improvement in the low in-service temperatures, although complementary rheological tests are required to confirm this assumption. 

## 4. Concluding Remarks

- Rheokinetic curves and rheological tests have shown epoxy binders present an enhanced behavior after 24 h mixing at 180 °C (particularly, at high epoxy concentration). Moreover, this improvement is able to continue under ambient curing for at least one month. As a result, the cured 4 wt % epoxy binder presents viscosity values at 60 °C, much higher than those obtained for the SBS binder.
- Temperature sweep tests in oscillatory shear reveal that, above 50 °C, this epoxy binder displays elastic properties similar to the reference rubbery binder.
- The improvement in the thermo-rheological properties occurred during either the mixing stage or the ambient curing. In this regards, FTIR and SARAs tests confirm a chemical modification through esterification/etherification reactions between the oxirane groups of BPA and the carbonyl groups available in aromatics and resins.
- New bitumen compounds are quantified in the asphaltenic fraction. Likewise, these high molecular weight compounds are more structured and ordered, as confirmed by the increase in the area corresponding to the fourth endothermic event in the nonreversing heat flow curves.
- Interestingly, in addition to the improved rheological response observed at 60 °C, all epoxy binders exhibit viscosity values, at 135 °C, lower than 3 Pa·s. According to AASHTO MP320, the compaction of their resultant asphalt mixes would be carried out successfully.

## Figures and Tables

**Table 1:** Carreau model parameters for the studied binders.

		$\eta_{0}$ *(Pa·s)*	λ *(s)*	*s*
	Blank	247 ± 7	0.08 ± 0.02	0.42 ± 0.01
	SBS	625 ± 9	14.89 ± 0.02	0.09 ± 0.01
Noncured binders	0.5 wt % BPA	311 ± 6	0.15 ± 0.02	0.27 ± 0.01
	1 wt % BPA	377 ± 6	0.24 ± 0.02	0.24 ± 0.01
	2 wt % BPA	480 ± 7	0.21 ± 0.02	0.36 ± 0.01
	3 wt % BPA	582 ± 9	0.40 ± 0.02	0.25 ± 0.01
	4 wt % BPA	667 ± 10	0.43 ± 0.02	0.29 ± 0.01
Cured binders	0.5 wt % BPA	347 ± 6	0.14 ± 0.02	0.38 ± 0.01
	1 wt % BPA	441 ± 7	0.19 ± 0.02	0.37 ± 0.01
	2 wt % BPA	530 ± 7	0.27 ± 0.02	0.32 ± 0.01
	3 wt % BPA	634 ± 9	0.40 ± 0.02	0.31 ± 0.01
	4 wt % BPA	815 ± 11	0.71 ± 0.02	0.28 ± 0.01

**Table 2:** Energy associated with the endothermic fourth event, ΔH_MDSC_, and characteristic temperature of the second event (T_MDSC_) obtained by MDSC for selected binders.

	ΔH_MDSC_ (J/g)	T_MDSC_ (°C)
Blank	0.34 ± 0.05	−2.5 ± 0.5
noncured 0.5 wt % BPA	0.95 ± 0.06	−3.2 ± 0.5
cured 0.5 wt % BPA	1.27 ± 0.06	−3.2 ± 0.6
noncured 4.0 wt % BPA	2.09 ± 0.07	−3.6 ± 0.5
cured 4.0 wt % BPA	2.41 ± 0.07	−4.7 ± 0.5

## Data Availability

The raw/processed data required to reproduce these findings cannot be shared at this time due to technical or time limitations.
